# E-Nose and GC-MS Reveal a Difference in the Volatile Profiles of White- and Red-Fleshed Peach Fruit

**DOI:** 10.3390/s18030765

**Published:** 2018-03-02

**Authors:** Rui Xin, Xiaohong Liu, Chunyan Wei, Chong Yang, Hongru Liu, Xiangmei Cao, Di Wu, Bo Zhang, Kunsong Chen

**Affiliations:** Zhejiang Provincial Key Laboratory of Horticultural Plant Integrative Biology/Laboratory of Fruit Quality Biology, Zhejiang University, Hangzhou 310058, China; ruixin@zju.edu.cn (R.X.); 21616053@zju.edu.cn (X.L.); chunyanwei@zju.edu.cn (C.W.); chongyang@zju.edu.cn (C.Y.); 11216045@zju.edu.cn (H.L.); xmcao@zju.edu.cn (X.C.); di_wu@zju.edu.cn (D.W.); akun@zju.edu.cn (K.C.)

**Keywords:** volatiles, fruit color, ripening, multivariate analysis, e-nose

## Abstract

First purchases of fruit are mainly dependent on aspects of appearance such as color. However, repeat buys of fruit are determined by internal quality traits such as flavor-related volatiles. Differences in volatile profiles in white- and red-fleshed peach fruit are not well understood. In the present study, peach cultivars with white- and red-fleshed fruit were subjected to sensory analysis using electronic nose (e-nose) to evaluate overview volatile profiles. Approximately 97.3% of the total variation in peach color-volatiles was explained by the first principle component 1 (PC1) and PC2. After analyzing sensory differences between peach fruit samples, 50 volatile compounds were characterized based on GC-MS. Multivariate analysis such as partial least squares discriminant analysis (PLS-DA) was applied to identify volatile compounds that contribute to difference in white- and red-fleshed peach fruit cultivars. A total of 18 volatiles that could separate peach fruit cultivars with different colors in flesh during ripening were identified based on variable importance in projection (VIP) score. Fruity note latone γ-hexalactone had higher contents in red-fleshed cultivars, while grassy note C6 compounds such as hexanal, 2-hexenal, (*E*)-2-hexenal, 1-hexanol, and (*Z*)-2-hexen-1-ol showed great accumulation in white-fleshed peach fruit.

## 1. Introduction

The peach is one of the most important fruit trees worldwide, and its fruit production was approximately 25 million tonnes in 2016 worldwide according to the Food and Agriculture Organization of the United Nations (FAO). China, Spain, and Italy produce approximately 70% of the peach crop in the world in 2016. The huge demand of peach fruit in human diet is due to its visual features, nutritional features, and unique flavour for consumption. Fruit color is the most important factor for consumers’ first-time purchases. The fruit flesh of most peach fruit is white or yellow in color. Meanwhile, there are a number of red or blood-fleshed peach cultivars grown in Asia and Europe [1]. For consumers, repeat buys of fruit are determined by internal quality traits such as flavor. Profiles of flavor-related volatiles have been thoroughly investigated for white and yellow-fleshed peach fruit [2,3,4,5] but not for the red ones.

Approximately 100 volatiles have been identified in peach fruit [2]. These chemicals could be grouped into aldehydes, alcohols, terpenoids, esters, and lactones based on compound structures. Volatiles are basically derived from precursors such as fatty acids, terpenes, and amino acids [6]. Peach fruit volatile compounds are highly dependent on genetic background. A great variation in volatiles was observed between 50 cultivars of peach fruit [3]. A study of yellow-fleshed peach ‘Redhaven’ and its white-fleshed mutant ‘Redhaven Bianca’ revealed marked differences in carotenoid levels and volatile profiles [4]. The yellow-fleshed peach fruit accumulated a high level of carotenoids and decreased levels of both isoprenoid and non-isoprenoid volatile [4]. Patterns of volatile components were applied to distinguish the Calanda peach cultivar from others, in order to protect the Designation of Origin and associated economic implication [7]. Moreover, significant changes in volatile profiles were observed during peach fruit development [8,9], ripening [10,11], and postharvest storage [12,13]. Correlation analysis revealed a relationship between volatiles and quality traits of peach fruit during ripening [14]. In general, fruity note esters and lactones tend to accumulate as peach fruit ripening, while grassy note aldehydes decrease. 

The formation of red-fleshed peach is due to the activation of anthocyanin biosynthesis by transcription factors such as NAC and MYB [1,15]. High levels of anthocyanins have been shown to reduce the incidence of chronic illnesses and to increase in protection against cancer [16]. Therefore, red-fruits with anthocyanins tend to be popular among consumers due to their healthy properties. However, components of flavor-related volatiles in red-fleshed fruit were not well understood. In the present study, eight peach cultivars with white- or red-fleshed fruit were collected. Considering great changes during peach fruit ripening, peach fruit were harvested at a similar ripe stage and then were subsequently stored at an ambient temperature for 3 d shelf-life to allow full ripening. E-nose was used to study over-view volatile profiles between white- and red-fleshed peach fruit. Volatile compounds were identified using GC-MS and were subject to multivariate statistical analysis such as non-supervised principle component analysis (PCA) and supervised partial least squares discriminant analysis (PLS-DA). Differences in volatile profiles between white- and red-fleshed peach fruit were observed, and compounds that contributed to separation of peach fruit with different colors were identified.

## 2. Materials and Methods

### 2.1. Plant Material and Sample Collection

Two groups of peach fruit samples were used in the present study: white- and red-fleshed cultivars. Peach fruit samples were collected from the Melting Peach Institute (Ningbo, China) and an orchard at Xian, China. Fruit was harvested at commercial mature stage and stored at 20 °C for 3 days to allow postharvest ripening. There are three replicates of five fruit each in the present study. In brief, there were eight peach cultivars and all of the peaches were stored for 0 d and 3 d, resulting in 48 samples for further analysis. Fruit ripening related quality indices such as total soluble solids (TSS) and firmness were described in Table 1. At each sampling time, slices of mesocarp were combined and frozen in liquid nitrogen and stored at −80 °C until use according to [10].

### 2.2. Fruit Ripening Evaluation

Total soluble solids (TSS) were measured by slicing both ends of each of the five samples of fruit for each biological replicate. Two drops of juice from each side were then applied to an Atago PR101α digital hand-held refractometer. Fruit firmness was measured according to method described [10]. A TA-XT2i Plus texture analyzer (Stable Micro System, Godalming, England) fitted with a 7.9 mm diameter head was used to evaluate peach fruit firmness at the equator. The rate of penetration was 1 mm s^-1^ with a final penetration depth of 10 mm and data are presented in Newton (N).

### 2.3. Volatile Compounds Analysis by E-Nose

The volatiles produced by peach fruit were evaluated using e-nose FOX 4000 (Alpha MOS, Toulouse, France) with three metal oxide sensor chambers equipped with 18 metabllic oxide sensors, according to the methods of our pevious study [12]. There are two types of sensors currently used: P & T sensors implemented in chambers A and B, and LY2 sensors used in chamber CL. Their specific names are LY2/LG, LY2/G, LY2/AA, LY2/GH, LY2/gCTl, 4LY2/gCT, T30/1, P10/1, P10/2, P40/1, T70/2, PA/2, P30/1, P40/2, P30/2, T40/2, T40/1, and TA/2. P & T sensors are metal oxide sensors based on tin dioxide (SnO_2_) (n-type semiconductor). Type T has the sensitive layer placed on a tube of aluminum, while the sensitive layer of type P is placed on a plain substrate. The LY2 sensors are metal oxide sensors based on chromium titanium oxide (Cr_2_ − xTixO_3_ + y) and on tungsten oxide (WO_3_).

Frozen flesh tissues were ground into powder by grinding in liquid nitrogen and transferred to a centrifuge tube containing 5 mL saturated sodium chloride solution. After mixing, 2 mL of homogenate were transferred into a 10 mL crimp-top vial and was then sealed with an aluminum gasket containing a PTFE/silica gel septum. In the course of e-nose analysis, crimp-top vials were warmed at 40 °C for 30 min in a dry bath heater. Headspace gas (2 mL) was injected into the Fox 4000 system and pumped into the sensor chamber with a constant rate of 150 mL∙min^−1^. The measurement phase lasted for 120 s for each sample, and the clean phase was 240 s. The 0 s and 100 s of response values of sensors in the e-nose were used as the e-nose data of each sample for further data analysis.

### 2.4. GC-MS Analysis of Volatiles

Volatile compounds were extracted using solid phase micro extraction (SPME) as previously reported [9]. Approximately 5 g of peach fruit tissue was ground into powder in liquid nitrogen and transferred to a 20 mL vial containing 3 mL of solution ethylene diamine tetraacetic acid (EDTA), and 3 mL of CaCl_2_. 2-octanol (0.7 mL∙min^−1^) was added as an internal standard before the vials were sealed. The vials were placed into a SPME autosampler (CombiPAL, Agilent Technologies, Palo Alto, CA, USA), coupled to an Agilent 7890A gas chromatograph (GC) and 5975C mass spectrometer (MS). Volatile analysis was performed using a DB-WAX column (30 m × 0.25 mm × 0.25 μm, J&W Scientific, Folsom, CA, USA). Helium was used as a carrier gas at 1.0 mL∙min^−1^. The temperature programme was set as follows: it started at 40 °C and was increased by 3 °C∙min^−1^ to 100 °C and then to 245 °C at 5 °C∙min^−1^. The column effluent was ionized by electron ionization (EI) at an energy of 70 eV with a transfer temperature of 250 °C and a source temperature of 230 °C. Volatiles were identified by comparison with the mass spectra of the National Institute of Standards and Technology (NIST) Mass Spectral Library (NIST-08) or with authentic standards when available (Sigma-Aldrich, St Louis, MO, USA). Relative quantification of compounds was carried out using the peak area of the internal standard as a reference based on total ion chromatogram (TIC).

### 2.5. Statistical Analysis

A completely randomized design was used in the experiments. Standard errors were calculated by Microsoft Excel. Principle component analysis (PCA) and discriminant function analysis (DFA) are used for volatiles components detected by e-nose using AlphaSoft version 11.0 (Alpha MOS, Toulouse, France). MULTI EXPERIMENT VIEWER (version 4.6.0) was applied for heatmaps of volatiles. Content of volatile compounds was subjected to partial least squares discriminant analysis (PLS-DA), and the value of variable importance in projection (VIP) was conducted by MetaboAnalyst 3.0 (http://www.metaboanalyst.ca/) with default parameter. PLS-DA model validation was performed by permutation test in which *p* < 0.05. VIP values exceeding 1.0 were selected as cutoff. Data were normalized using ‘Autoscaling’ in the metaboAnalyst program. The pearson correlation between e-nose sensors response and volatile content was analyzed using Origin 8.0 (Microcal Software Inc., Northampton, MA, USA). 

## 3. Results

### 3.1. Response of E-Nose to Peach Fruit

**Figure 1:**
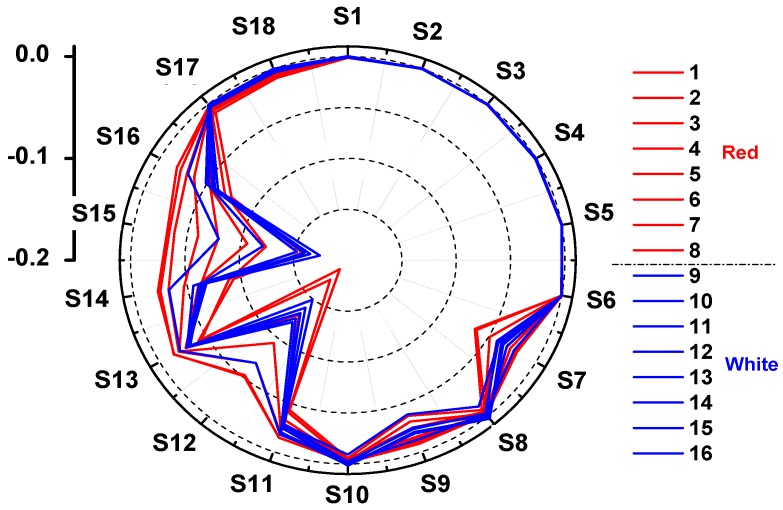
Polar plots of the fingerprints (the maximum or minimum response values) of typical peach samples. S1 to S18 means 18 sensors equipped by e-nose. Names of peach sample from 1 to 16 are shown in Table 1.

The average e-nose responses (the maximum response values) of the 18 sensors (S1–S18) for peach samples are shown in a polar plot in Figure 1. The response values of all sensors were jointed by straight lines. The values at the top-left corner of the polar plot are the scale of the sensor response produced by the e-nose against peach fruit samples. Sensors 1 to 6 represented very weak e-nose responses that were almost equal to 0 (Figure 1). Similar low values were also observed for sensors 8, 10, 17, and 18. The remarkable high response values were detected for e-nose sensors 12 and 15 (Figure 1), followed by sensors 14, 16, 7, 9, 11, and 13. Different response values between red-fleshed and white-fleshed peach fruit indicated potential application of e-nose in discriminating among different samples of peach fruit.

### 3.2. Classification of Peach Fruit Samples with Different Colors by E-Nose

**Figure 2:**
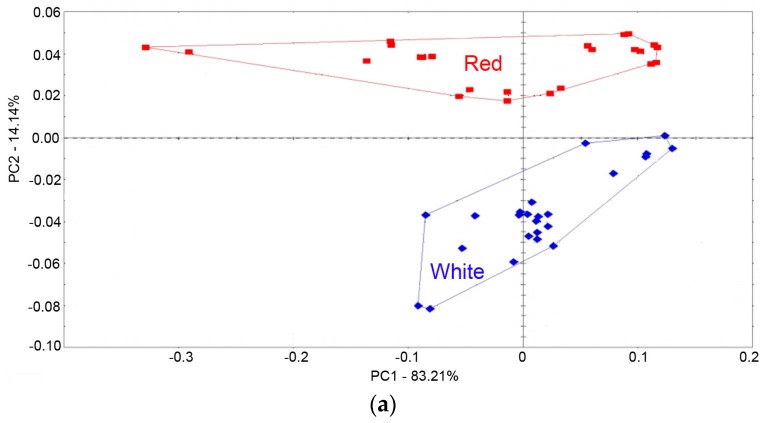

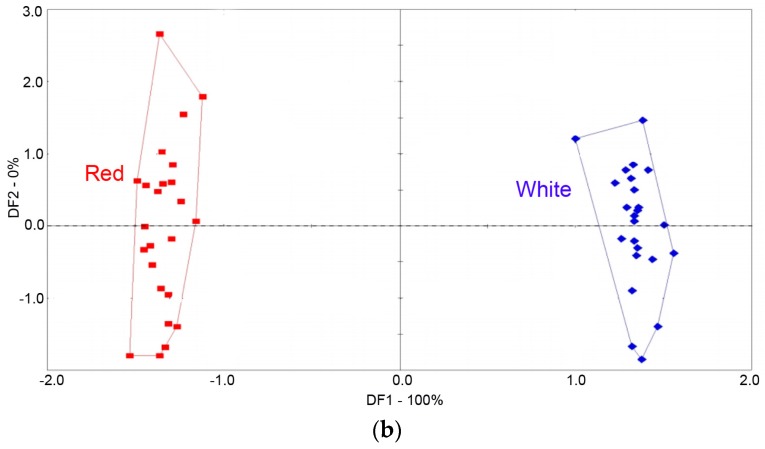
Multivariate analysis of peach fruit volatile compounds measured by e-nose. (**a**) PCA score plot, (**b**) DFA score plot.

To detect potential differences among volatiles produced by different samples of peach fruit juice, PCA and DFA were used to check this capability of e-nose in assigning peach fruit samples to a specific group. PCA analysis showed that the PC1 and PC2 explained over 97.3% of the total variation in the peach color-volatiles relationship (Figure 2), indicating that information from e-nose was almost included in the first two PCs. Red-fleshed peach samples were mainly distributed on the positive side of the PC2, while white-fleshed samples were mainly located on the negative scores on the PC2. For DFA, the first two factors explained 100% variance rates. In agreement with PCA results, all peach fruit samples from red-fleshed group were located at the left side of DF1, while white-fleshed samples were distributed at the right side of DF1 (Figure 2).

PLS-DA is a classification technique based on partial least squares (PLS). The general principle is that the regression model between the independent variable X and the categorical variable Y was established based on the information of sample classification in the process of variables extraction, and then the samples were classified according to the PLS prediction value of samples. In this study, the PLS-DA model was established between the encoded binary variety numbers and the electronic nose data. In this process of PLS-DA, the Y values of peach samples were set 1 and −1 for red peach and white peach, respectively, and then the regression of Y onto X was calculated. 

To further test the capability of e-nose in the classification of two different colored peach samples, the model of PLS-DA was carried out through analyzing the e-nose data. It was worth noting that the calibrated PLS model must be evaluated for its validity, and the validation method of segmented cross-validation was applied for the validation purpose. In the process of segmented cross-validation, several samples were used as the calibration set, and the remaining samples were used as the validation set, until all samples were validated. The process is looped until all samples were validated. In this study, all peach samples were carried out for calibration and validation. The accuracy of modeling set and calibration set in the PLS-DA classification model was 97.9% and 97.9%, respectively. The results indicate that it is possible to use the PLS-DA classification model to classify the red and white peaches by analyzing the volatile compounds measured by e-nose. 

### 3.3. Volatiles Play a Role in Discriminating Peach Fruit Samples

**Figure 3:**
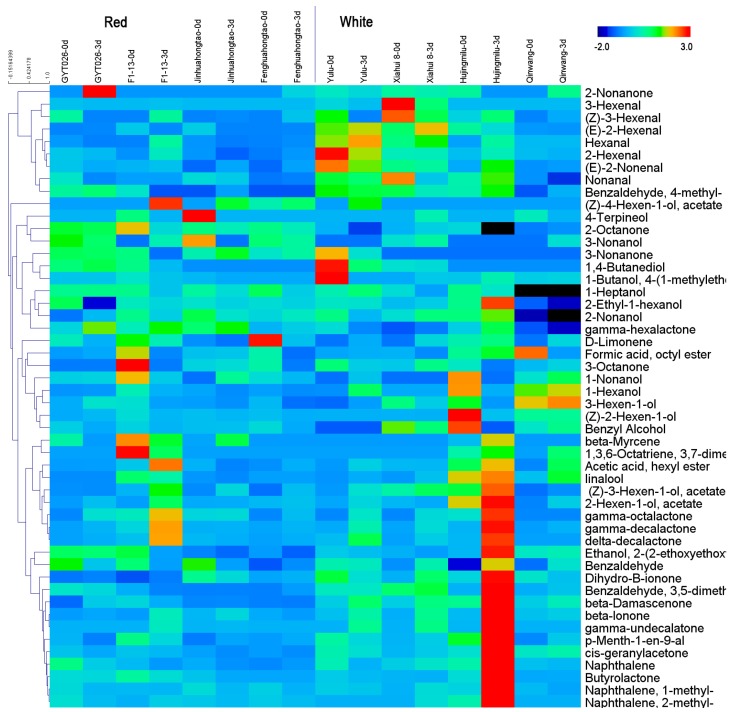
Heatmap display of peach fruit volatiles measured by GC-MS.

Multivariate analysis of the response of the e-nose sensors showed that red-fleshed and white-fleshed peach samples could be classified, indicating possible difference in volatile compounds released by these two peach groups. Therefore, the traditional technique GC-MS was applied to detect difference in concentrations of individual volatile component, although this method is time-consuming, complicated, and costly. A total of 50 volatiles were detected in a total of 48 peach samples, including C6 compounds, esters, lactones, terpenes, norisopernoids, ketones, alcohols, phenypropanoids, and others. It was somewhat surprising that variations occurred in volatile contents by as much as approximately 1045-fold across peach samples (Table 2). Significant difference in fruit volatile contents was observed in peach cultivars at different ripening stages (Figure 3).

**Figure 4:**
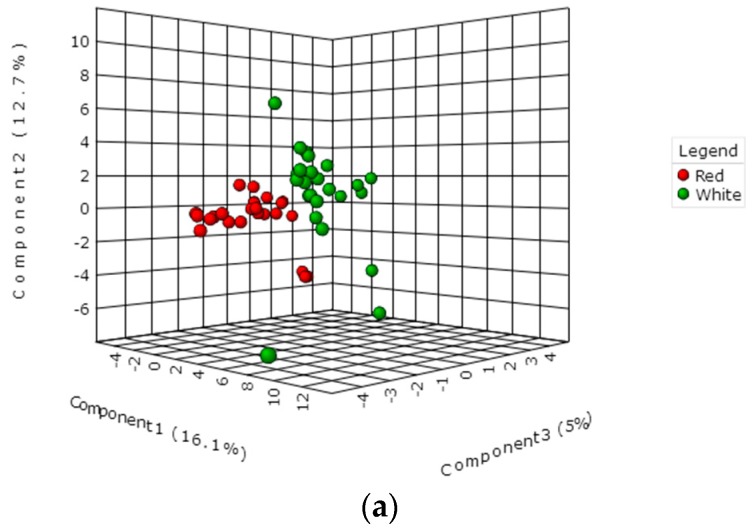

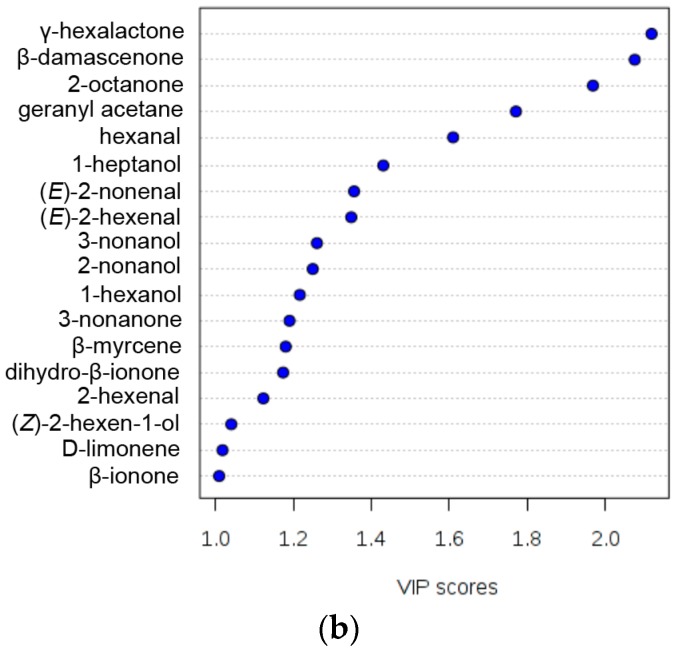
Multivariate analysis of peach fruit volatile compounds measured by GC-MS. (**a**) PLS-DA score plot, (**b**) volatiles ranked by VIP scores (blue dots).

PLS-DA is a supervised multivariate analysis method, and it is widely used for classification and biomarker selection in metabolomics. To compare differences in volatile compounds between peach samples with two different colors, volatiles were subjected to PLS-DA analysis. Statistical significance of the PLS-DA model was evaluated using a permutation test. The test showed that discrimination between fruit samples was statistically significant (*p* < 0.01). In PLS-DA score plot, red-fleshed peach fruit were separated from white-fleshed samples (Figure 4a). These results indicated significant differences in volatile compounds of peach samples with two colors. 

To identify volatile compounds that could explain separation of peach fruit with two colors, values of variable importance in projection (VIP) were calculated. VIP is used to explain the weight of the independent variable in explaining the dependent variable, which is usually considered to have important roles in the PLS-DA discriminant process when values exceed 1.0. A total of 18 volatile compounds with VIP score > 1.0 were identified (Figure 4b), indicating their importance in separation of peach fruit samples as shown in PLS-DA score plot (Figure 4b). Among these volatiles, γ-hexalactone had the highest VIP value of 2.12, implying that this compound is the most important variable as a potential marker for ripening different peach fruit samples with two colors.

**Figure 5:**
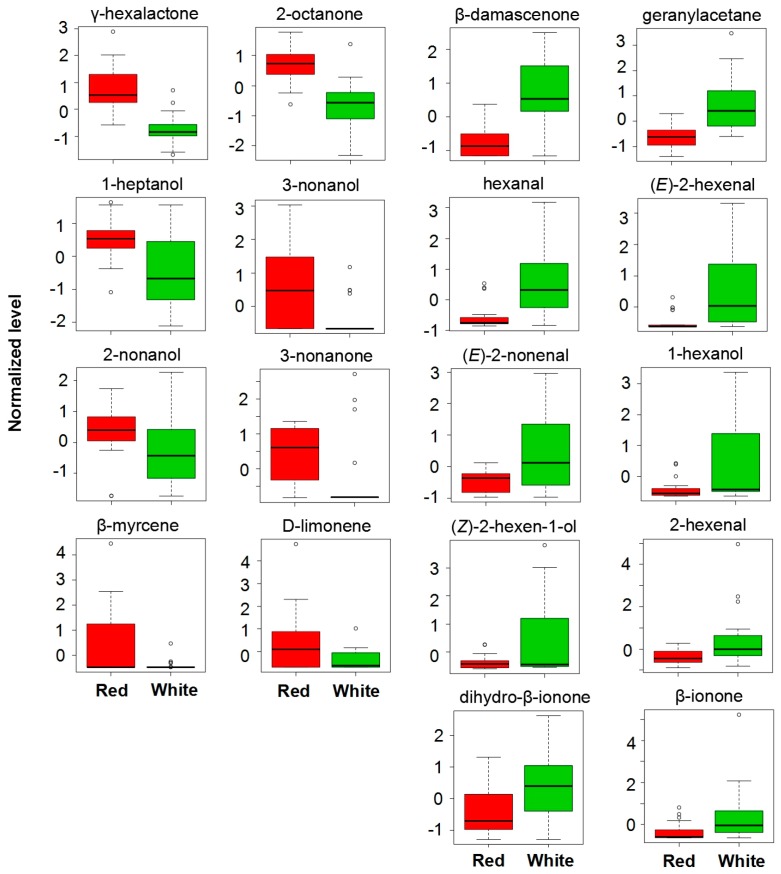
Box plot of volatile contents with VIP score > 1 in red and white peach fruit.

For γ-hexalactone, high concentration was observed in red-fleshed peach fruit when compared to that of white-fleshed samples (Figure 5). Red-fleshed peach fruit also produced higher content of terpenes such as β-myrcene and D-limonene, alcohols such as 1-heptanol, 3-nonanol, and 2-nonanol. In contrast, some other volatile compounds with VIP value above 1.0 had higher contents in white-fleshed peach fruit. For example, white-fleshed peach fruit accumulated more contents of C6 compounds such as hexanal, 2-hexenal, (*E*)-2-hexenal, 1-hexanol, and (*Z*)-2-hexen-1-ol (Figure 5). Similar high contents in white-fleshed were also observed for volatile norisoprenoids such as β-damascenone, Dihydro-β-ionone, geranyl acetane, and β-ionone.

## 4. Discussion

Peaches are one of the most important horticultural crops, whose fruit is widely produced in Asia, Europe, and American. Recently, red-fleshed peach fruit has received attention from the human health advocates due to its high antioxidant capacity of anthocyanin [1]. In the present study, different volatile profiles of red-fleshed peach fruit were observed relative to white-fleshed ones.

Eight cultivars of peach fruit were used to investigate differences in volatile profiles between white- and red-fleshed types, because volatile components are affected by their genetic backgrounds [3]. Considering that volatile compounds are affected by fruit ripening stages [10], two ripening stages of peach fruit were selected for volatile profile analysis, including commercial maturity stage at harvest day and subsequent 3 d shelf-life for artificial ripening. Based on texture of fruit during ripening, peach fruit consists of melting and nonmelting types, the former usually softening completely after 3 d shelf-life, while the latter maintains high firmness level after harvest storage (Table 1). To further investigate volatile differences among white- and red-fleshed peach fruit samples, each color peach included melting and non-melting types in the present study. To our knowledge, this is the first study to explore volatile profiles of peach fruit samples between white-fleshed and red-fleshed cultivars, including different ripening stages and melting types.

E-nose is an electronic system that consists of chemical sensor arrays and pattern recognition methods, which tries to imitate the human sense of smell. Sensory analysis by a panel of experts is a costly process, while e-nose possesses ease of operation and requires only a short time to be analysed for tested odorants [17]. Therefore, e-nose was applied as a fast and qualitative method for studying peach fruit flavor quality [12,18,19]. In the present study, differences among volatile profiles between red-fleshed and white-fleshed peach fruit were detected using e-nose. The PCA and DFA results showed that the e-nose could discriminate peach fruit samples from two different flesh colors with reasonable accuracy. The model of PLS-DA further showed that e-nose could classify two different colored peach samples. These results indicated that the e-nose provided a rapid way of telling the difference in aroma quality between red-fleshed and white-fleshed peach fruit. Moreover, this observation prompted us to further study volatile profiles of peach fruit samples with two colors by using GC-MS, although the preparation of the sample is different for these two techniques.

Volatiles were used to create a classification function that distinguished the Calanda peach cultivar from other cultivars in Europe [7]. In the present study, the contents of volatiles varied greatly in peach fruit with different colors and were subjected to multivariate analysis. In agreement with the classification of peach fruit by e-nose, PLS-DA plot (Figure 4a) showed that volatile components are strongly correlated with flesh color. A total of 18 volatiles that could explain the separation of peach fruit samples with two colors were identified based on VIP scores. The highest score was observed for γ-hexalactone, which accumulated higher content in peach fruit samples with red flesh when compared to that of white-fleshed fruit. The sensory analysis of peach fruit showed that γ-hexalactone made the highest positive contributions to the intensity of the ripe fruit aroma [5]. Moreover, higher concentrations of volatile compounds positively related to peach fruit sensory acceptance were also observed in red-fleshed peach fruit. For example, woody note volatile β-Myrcene was able to drive consumer demand, while 1-Heptanol was positive to fruity and peach-like sensory [5]. In contrast to volatiles described above, grassy note aroma accumulated high in white-fleshed peach fruit, including volatile C6 compounds such as (*E*)-2-hexenal, 2-hexenal, 1-hexanol, and (*Z*)-2-hexen-1-ol. Descriptive sensory analysis revealed that the unpleasant flavor of peach fruit was highly associated with β-damascenone [20], which had a higher concentration in white-fleshed peach fruit. These results indicated that red-fleshed peach fruit may have a stronger fruity aroma and sensory intensity than white-fleshed fruit. Significant difference in volatiles between peach fruit cultivars and different ripening stages were observed (Table 2, Figure 3), in agreement with previous studies [3,10]. Although significant differences in volatiles were observed as fruit ripening, PLS-DA results revealed significant separation between peach cultivars due to flesh color. These results indicated that genetic background plays a major role in affecting peach fruit volatile profiles.

Present observations showed that both e-nose and GC-MS analysis could be applied to detect the difference in volatile profiles between peach cultivars due to flesh color. The application of e-nose provided an overview volatile profile of fruits and improved the effectiveness and the efficiency of the process. The progress of e-nose application in fruit aroma evaluations was reviewed, and the potential for future development were proposed [21]. GC-MS analysis allowed for the detection of all volatile compounds emitted by fruits, and for the precise identification of specific volatiles that contributed to differences between samples. Compared to the e-nose, GC-MS is a traditional and time-consuming instrument. The purpose of this project or study should define whether one or both together was emitted by fruits or other agricultural products in the process of detection volatiles. 

## 5. Conclusions

In summary, peach fruit with different flesh colors varies in volatile profiles at ripe stages. E-nose revealed different sensory qualities between white- and red-fleshed peach fruit, and GC-MS unraveled at least 18 volatiles that contribute to flavor difference. Red-fleshed peach fruit accumulated more fruity or peach like volatiles compared to that produced by white-fleshed fruit. Volatiles showed different concentrations in white- and red-fleshed peach, such as lactones and terpenoids. Candidate genes contributed to differences in volatile profiles between peach fruit with different colors that are required to be characterized in future.

## Figures and Tables

**Table 1:** Peach fruit samples used in the present study.

Code	Peach Cultivar	Flesh Color	Sample Description	TSS ^1^ (Brix)	Firmness (N)
1	GYT026	red	Harvest day	12.51 ± 0.65	13.23 ± 1.77
2			3 d shelf-life	12.84 ± 0.51	3.54 ± 0.2
3	F1-13	red	Harvest day	10.42 ± 0.41	7.68 ± 1.49
4			3 d shelf-life	11.73 ± 0.28	3.15 ± 0.09
5	Fenghuahong	red	Harvest day	11.88 ± 0.83	15.64 ± 2.26
6			3 d shelf-life	11.31 ± 0.75	4.62 ± 0.43
7	Jinghuahong	red	Harvest day	7.92 ± 0.51	30.58 ± 2.65
8			3 d shelf-life	8.74 ± 0.46	26.17 ± 1.89
9	Yulu	white	Harvest day	14.22 ± 0.32	29.38 ± 2.76
10			3 d shelf-life	15.54 ± 0.47	5.61 ± 0.38
11	Xiahui 8	white	Harvest day	16.44 ± 0.41	32.45 ± 3.22
12			3 d shelf-life	15.99 ± 0.3	15.82 ± 2.22
13	Hujingmilu	white	Harvest day	13.61 ± 0.54	42.36 ± 1.58
14			3 d shelf-life	14.72 ± 0.62	4.76 ± 0.75
15	Qinwang	white	Harvest day	11.88 ± 0.36	21.28 ± 3.17
16			3 d shelf-life	13.58 ± 0.57	28.33 ± 2.15

^1^ Total soluble solids.

**Table 2:** Observed variation in volatile compounds (ng/g∙FW) in peach fruit.

	Red				White			
	High	Low	Fold Difference	Average	High	Low	Fold Difference	Average
Hexanal	378.82	9.12	41.52	70.56	941.94	0.90	1045.44	355.74
3-Hexenal	6.11	2.71	2.25	3.96	314.32	0.50	623.40	78.87
(*Z*)-3-Hexenal	5.28	0.14	36.65	3.15	18.84	1.13	16.71	6.27
2-Hexenal	175.37	11.18	15.68	73.14	705.87	11.94	59.12	187.58
(*E*)-2-Hexenal	72.90	0.98	74.11	32.32	349.49	5.97	58.58	121.68
1-Hexanol	117.48	5.10	23.05	31.82	451.44	2.61	172.65	135.07
(*Z*)-2-Hexen-1-ol	32.77	2.47	13.25	11.77	263.28	2.15	122.49	45.29
3-Hexen-1-ol	9.46	0.66	14.39	5.48	36.76	1.98	18.54	9.87
Nonanal	19.66	5.51	3.57	11.41	52.97	1.49	35.44	21.12
(*E*)-2-Nonenal	6.51	1.62	4.02	3.52	20.01	1.36	14.72	9.51
Acetic acid, hexyl ester	100.40	2.37	42.43	17.75	104.05	3.92	26.54	25.79
(*Z*)-3-Hexen-1-ol, acetate	151.03	13.24	11.40	53.82	246.43	10.74	22.95	88.78
(*Z*)-4-Hexen-1-ol, acetate	24.71	6.77	3.65	13.28	32.97	2.99	11.02	13.37
2-Hexen-1-ol, acetate	27.84	1.23	22.69	7.71	64.10	1.59	40.20	14.76
Formic acid, octyl ester	18.75	3.70	5.06	6.67	23.36	3.76	6.22	7.53
β-Myrcene	14.92	0.28	53.43	5.93	7.83	1.01	7.78	2.74
D-Limonene	32.84	1.28	25.65	9.26	12.73	1.15	11.10	6.40
1,3,6-Octatriene, 3,7-dimethyl-	9.47	2.77	3.42	5.08	4.34	2.16	2.01	2.98
Linalool	146.30	1.75	83.75	40.96	407.75	2.33	175.07	93.59
4-Terpineol	9.31	1.63	5.72	4.15	2.01	1.54	1.30	1.77
p-Menth-1-en-9-al	6.69	0.88	7.57	2.90	15.00	0.76	19.65	5.44
β-Damascenone	3.61	0.52	6.99	1.58	15.72	2.13	7.38	5.64
Dihydro-β-ionone	13.51	1.50	9.04	4.99	31.72	2.50	12.67	10.63
cis-geranylacetone	4.72	0.93	5.09	2.36	24.58	2.32	10.58	7.28
β-Ionone	2.53	0.14	17.64	0.94	16.54	0.34	48.13	2.62
3-Octanone	6.18	1.03	6.00	2.75	3.63	0.45	7.99	1.39
2-Octanone	460.72	251.74	1.83	328.41	280.70	41.90	6.70	208.58
3-Nonanone	6.97	3.17	2.20	5.38	11.33	3.50	3.23	8.02
2-Nonanone	11.61	1.29	8.97	6.03	3.17	1.17	2.71	2.52
1-Heptanol	38.13	28.79	1.32	31.95	42.19	20.00	2.11	28.95
2-Ethyl-1-hexanol	15.11	0.81	18.54	10.53	25.32	3.96	6.40	11.48
3-Nonanol	37.86	11.04	3.43	21.34	16.19	11.53	1.40	13.79
2-Nonanol	12.96	4.89	2.65	7.79	13.39	0.57	23.48	7.25
Ethanol, 2-(2-ethoxyethoxy)	30.94	4.53	6.82	13.92	45.90	9.94	4.62	16.98
1-Nonanol	6.80	0.91	7.44	2.61	6.85	1.52	4.50	4.06
Benzyl Alcohol	3.03	0.93	3.25	1.46	7.05	1.38	5.13	3.73
1,4-Butanediol	3.00	0.17	17.70	1.54	7.47	0.77	9.69	2.00
1-Butanol, 4-(1-methylethoxy)	6.93	0.40	17.37	2.24	68.89	1.82	37.86	7.58
Benzaldehyde	20.64	4.59	4.50	10.77	23.96	3.88	6.17	10.69
Benzaldehyde, 4-methyl	5.07	1.13	4.50	2.79	5.02	1.39	3.62	3.67
Benzaldehyde, 3,5-dimethyl	5.49	0.33	16.50	2.00	16.75	1.43	11.75	6.30
Butyrolactone	14.01	2.29	6.11	6.01	46.82	2.01	23.24	7.10
γ-hexalactone	10.26	2.39	4.28	5.54	6.76	0.14	46.73	2.92
γ-octalactone	4.13	0.31	13.18	1.47	4.12	0.53	7.79	1.81
γ-decalactone	142.67	1.09	130.91	25.28	164.70	0.38	431.31	37.67
δ-decalactone	28.90	0.60	48.44	5.85	35.32	1.22	29.07	10.34
γ-undecalatone	7.00	0.05	135.219	2.39	49.24	0.40	122.78	12.70
Naphthalene	6.91	1.57	4.39	3.12	19.15	2.48	7.73	5.11
Naphthalene, 1-methyl	5.71	0.66	8.67	2.06	42.03	0.59	71.57	5.88
Naphthalene, 2-methyl	2.74	0.32	8.53	1.16	29.61	1.57	18.90	8.69
